# Apelin13/APJ promotes proliferation of colon carcinoma by activating Notch3 signaling pathway

**DOI:** 10.18632/oncotarget.21904

**Published:** 2017-10-13

**Authors:** Tong Chen, Ning Liu, Guang-Meng Xu, Tong-Jun Liu, Ying Liu, Yan Zhou, Si-Bo Huo, Kai Zhang

**Affiliations:** ^1^ Gastrointestinal Surgery Department, The China-Japan Union Hospital of Jilin University, Jilin, China; ^2^ Colorectal Surgery Department, The Second Hospital of Jilin University, Jilin, China; ^3^ General Surgery Department, The First Hospital of Jilin Province Academy of Traditional Chinese Medicine, Jilin, China

**Keywords:** Apelin/APJ, Notch3, colon carcinoma, cancer proliferation

## Abstract

**Background:**

The link between Apelin (APL)/APL receptor (APJ) and Jagged (JAG)/Notch signaling pathways in colorectal cancer (CRC) has been poorly investigated. APL/APJ system, a potent angiogenic factor, is up-regulated in a variety of cancers. It contributes to tumor angiogenesis, and correlates with progression of malignancy. JAG/Notch signaling also contributes to progression, proliferation and metastasis of multiple cancers, including CRC. Here we tested the hypothesis that APL/APJ system promotes CRC proliferation by up-regulating Notch3, thus allowing further binding of JAG1 to Notch3.

**Materials and Methods:**

We used a variety of methods including Western blot, RT-qPCR, gene silencing, ELISA, immunofluorescence staining, to investigate the interaction between APL/APJ system and Notch3 signaling pathway in both surgically-resected specimens and CRC cell line LS180.

**Results:**

We show that the expression of APL13, APJ, and Notch3 is elevated in CRC. We further demonstrate that APL13 can be secreted into culture media of LS180 cells, suggesting the existence of autocrine loop in CRC. Moreover, we found that APL13 stimulated expression of Notch3. Finally, we found that inhibition of either APJ or Notch3 prevents proliferation of LS180 cells.

**Conclusions:**

Our results suggest that APL13/APJ and JAG1/Notch3 signaling pathways are linked in CRC. These findings provide a new direction to the efforts targeting effective therapeutic and management approaches in the treatment of CRC.

## INTRODUCTION

Apelin (APL) signaling pathway was recently shown to participate in many physiological and pathophysiological processes [1]. Apelin exists in several isoforms including APL-12, -13, -17, and -36 [2]. Apelin receptor (APJ) is a member of the G protein coupled receptor family, which remained orphan until the discovery of APLs [3]. The well-documented roles of APL signaling are closely related to the expression of APJ in hypothalamus, blood vessels, and heart. During blood vessel formation, the level of APJ mRNA in endothelial cells is very high [4, 5]. This endothelial expression is associated with the mitogenic action of apelin on these cells, which is necessary for the spread of vascular network. Collectively, the existing data suggest that APLs display the essential properties required for angiogenic peptides. Indeed, APL12 is capable of increasing myocardial contractility, and APL13 contributes to regulation of glucose homeostasis and prevents ischemia/reperfusion injury [5, 6]. Of note, APL13 is the most active form binding to APJ receptor [3]. By using mice *in vivo* model for studying the role of APL13 in the repair of postmyocardial infarction, Li and co-workers showed that the expression of CXCL12/CXCR-4 was up-regulated and phosphorylation of Akt and eNOS was considerably increased in animals receiving APL13 treatment. Interestingly, the treatment also up-regulated VEGF and Jagged-Notch3 expression in ischemic hearts [7].

Notch signaling pathway plays a critical role in intestinal epithelial stem/progenitor cell self-renewal and differentiation. To date, four Notch receptors (Notch 1–4) and five Notch ligands (Delta-like 1 [DLL1], DLL3, DLL4, Jagged-1 (JAG1) and JAG2) have been reported [8]. JAG1, like the other ligands, interacts with Notch receptors to activate the cleavage of Notch receptors by c-secretase leading to the release of Notch intracellular domain (NICD) [8, 9]. Subsequently, NICD forms a complex with a transcriptional regulator in the nucleus to induce transcription of target genes, such as HES gene family.

It has been shown that Notch signaling is strongly activated in primary human colorectal cancer (CRC) and has an important role in the onset and progression of CRC through mediation of apoptosis, proliferation, angiogenesis and cell migration [10–14]. Recent reports have also indicated that JAG1 mediates the activation of Notch signaling in CRC and induces CRC progression [15–19]. In addition, it was reported that a strong correlation exists between high JAG1 expression, KRAS status, and prognosis of CRC [9]. The latter study also revealed that low expression of E-cadherin plays an additive role for poor prognosis associated with high JAG1 expression in CRC, providing further clues for potential mechanisms of complex regulation of JAG1 expression and JAG1-Notch pathway-induced cancer development.

Given that APL13 can stimulate JAG1/Notch3 signaling in ischemic hearts [7], in this study we investigated whether the treatment with APL13 activates JAG1/Notch3 to promote cancer proliferation in CRC. Our data suggest that the APL/APJ system can be activated in autocrine manner, resulting in up-regulation of Notch3 expression, leading to proliferation of CRC.

## RESULTS

### APL13, APJ, and Notch3 are overexpressed in colon adenocarcinoma tissues

**Figure 1:**
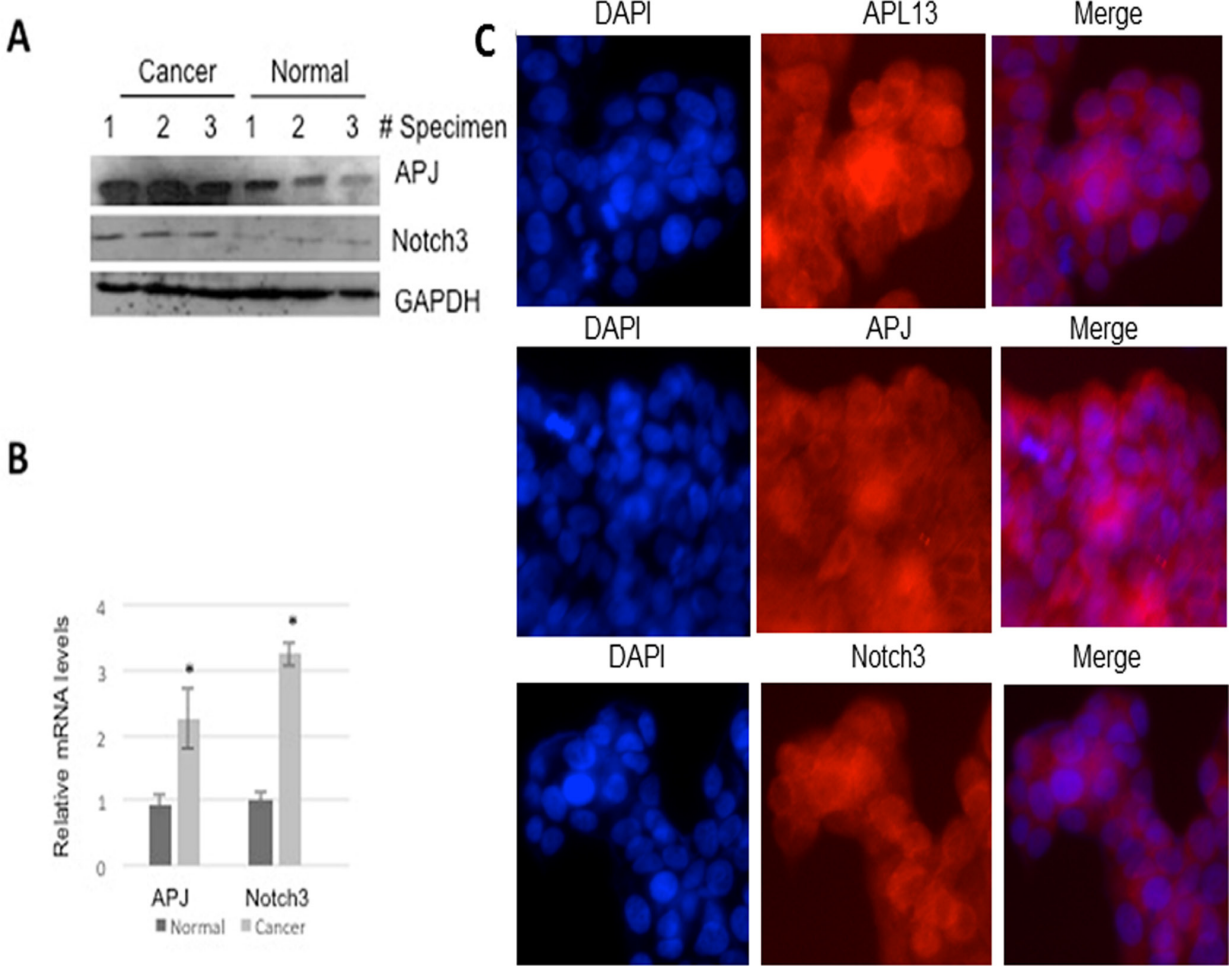
APL13, APJ, and Notch3 are overexpressed in human colon adenocarcinoma (**A**) Western blot analysis of resected tissues from cancerous or adjacent normal tissues of colon adenocarcinoma. (**B**) RT-qPCR were used to assess mRNAs of APJ or Notch3 from these tissues (*N* = 3). (**C**) Immunofluorescence staining of LS180 colon carcinoma cell line with use of antibodies as indicated (*N* = 3). ^*^P < 0.01.

To assess the expression of these genes *in vivo*, we used Western blot and RT-qPCR for determining their protein and mRNA levels in three individual surgically resected specimens of colon carcinoma (stage III) with adjacent normal tissues as controls. As shown in Figures 1A and 1B, the levels of APJ and Notch 3 protein and mRNA levels were significantly increased in colon carcinoma in contrast to those in the adjacent normal tissues. In order to further confirm that colon carcinoma cell line express these genes, we used human colon carcinoma cell line LS180 as the *in vitro* model. Cells were cultured for 24 h, and then subjected to immunofluorescence staining. Figure 1C shows that LS180 cells constitutively expressed these 3 genes. Taken together, the data suggest that APL13/APJ-Notch3 signaling pathway plays a role in proliferation of colon carcinoma.

### APL13 is secreted from colon carcinoma

**Figure 2:**
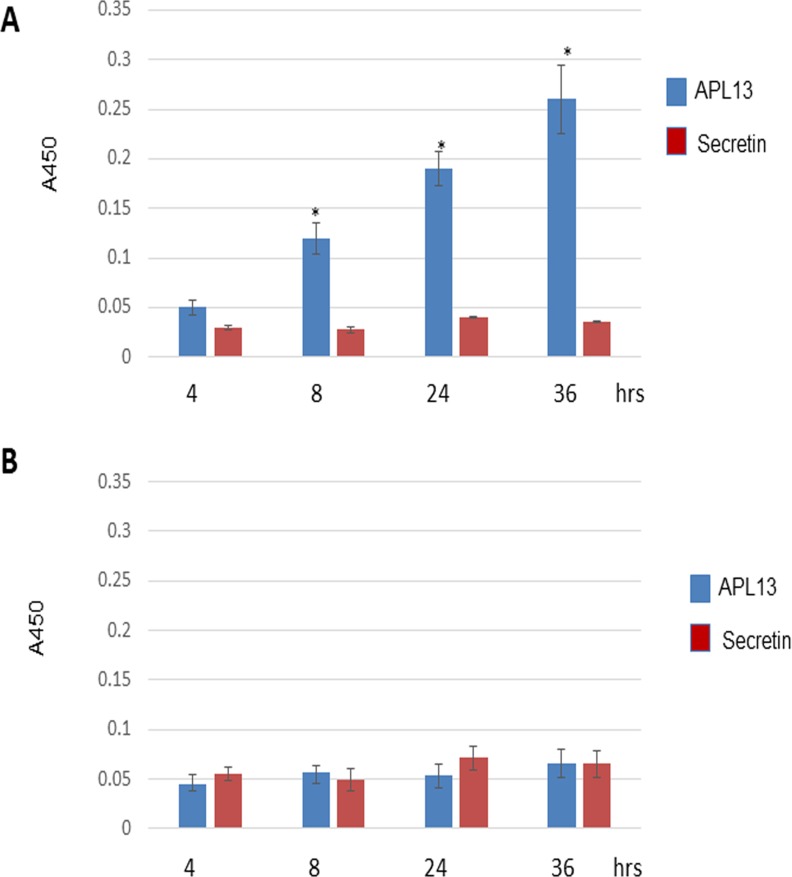
LS180 cells secrete APL13 (**A**) Frozen LS180 cells were defrosted and cultured in RMPI1640 and 10% FBS for indicated time periods. The culture media were sampled for ELISA assay. (**B**) Frozen HEK293 cells were defrosted and cultured in RMPI1640 and 10% FBS for indicated time periods. The culture media were sampled for ELISA assay. *N* = 3. ^*^P < 0.01.

The constitutive expression of APL13 in colon carcinoma suggests that APL can act on APJ in an autocrine manner, as previously described [20]. In order to test whether colon carcinoma can secrete APL13, we cultured LS180 cells in RPMI1640 and 10% FBS. The media were sampled at 4, 8, 24, or 36 h and subjected to ELISA assay. We also used secretin, a specific protein secreted from small intestines, as a negative control. As shown in Figure 2, APL13 was secreted by LS180 cells in a time-dependent manner. As expected, secretin was not detected in the media even after 36 h of culturing. HEK293 cells secreted no to minimal APL13 or secretin (Figure 2B). These data support that APL13 can stimulate APJ on the basis of autocrine loop.

### APL13/APJ activates Notch3

**Figure 3:**
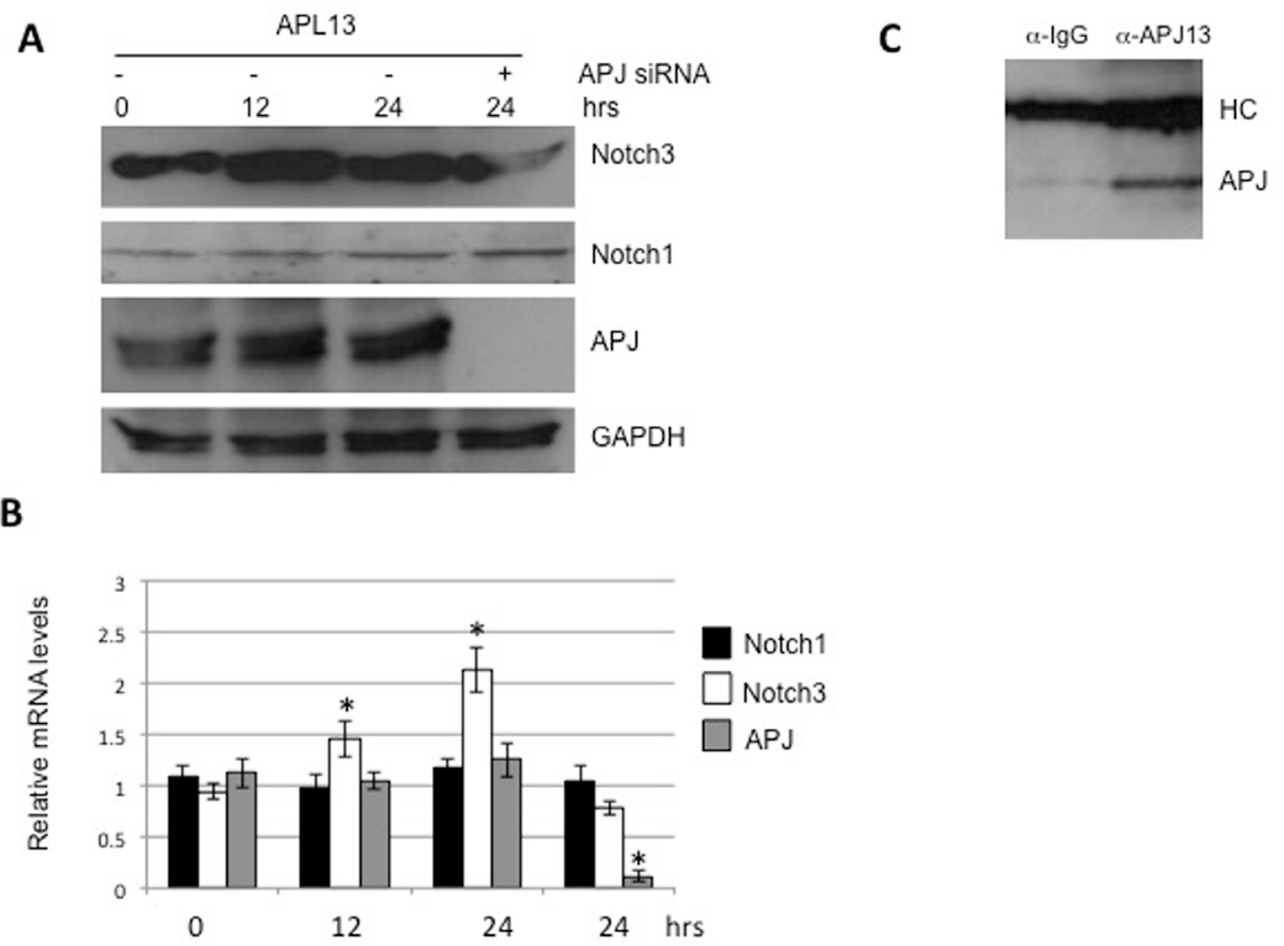
APL13 induces production of Notch3 Frozen LS180 cells were defrosted and cultured in RMPI1640 and 10% FBS for 24 h. The media was changed to fresh one, and the cells were treated with APL13 for the time period as indicated. The whole lysates were subjected to Western blot analysis (**A**) or extracted for isolation of total RNA, followed by RT-qPCR analysis (**B**). (**C**) Frozen LS180 cells were defrosted and cultured in RMPI1640 and 10% FBS for 24 h. The cells were lysed with RIPA buffer and immunoprecipitated with normal rabbit IgG (IgG) or anti-APL13 antibodies, as indicated. The immunoprecipitates were subjected to Western blot analysis using antibody to APJ. *N* = 3. ^*^P < 0.01.

As reported previously, APL13/APJ system causes up-regulation of Notch3 in post-myocardial infarction [7]. In order to determine whether such a system exerts similar effects in colon carcinoma, we treated LS180 cells with APL13. The culture media for the cells was changed to a fresh one to minimize the effect of endogenous APL13. As shown in Figures 3A and 3B, APL13 stimulated the production of Notch3, but not Notch1, at both protein and mRNA levels in a time-dependent manner. In addition, the depletion of APJ abolished this up-regulation. Interaction of APL13 and APJ was confirmed by immunoprecipitation (Figure 3C). These findings suggest that APL13/APJ system stimulates the proliferation of colon carcinoma by up-regulating Notch3.

### JAG1/Notch3 signaling pathway activates Hes in colon carcinoma

**Figure 4:**
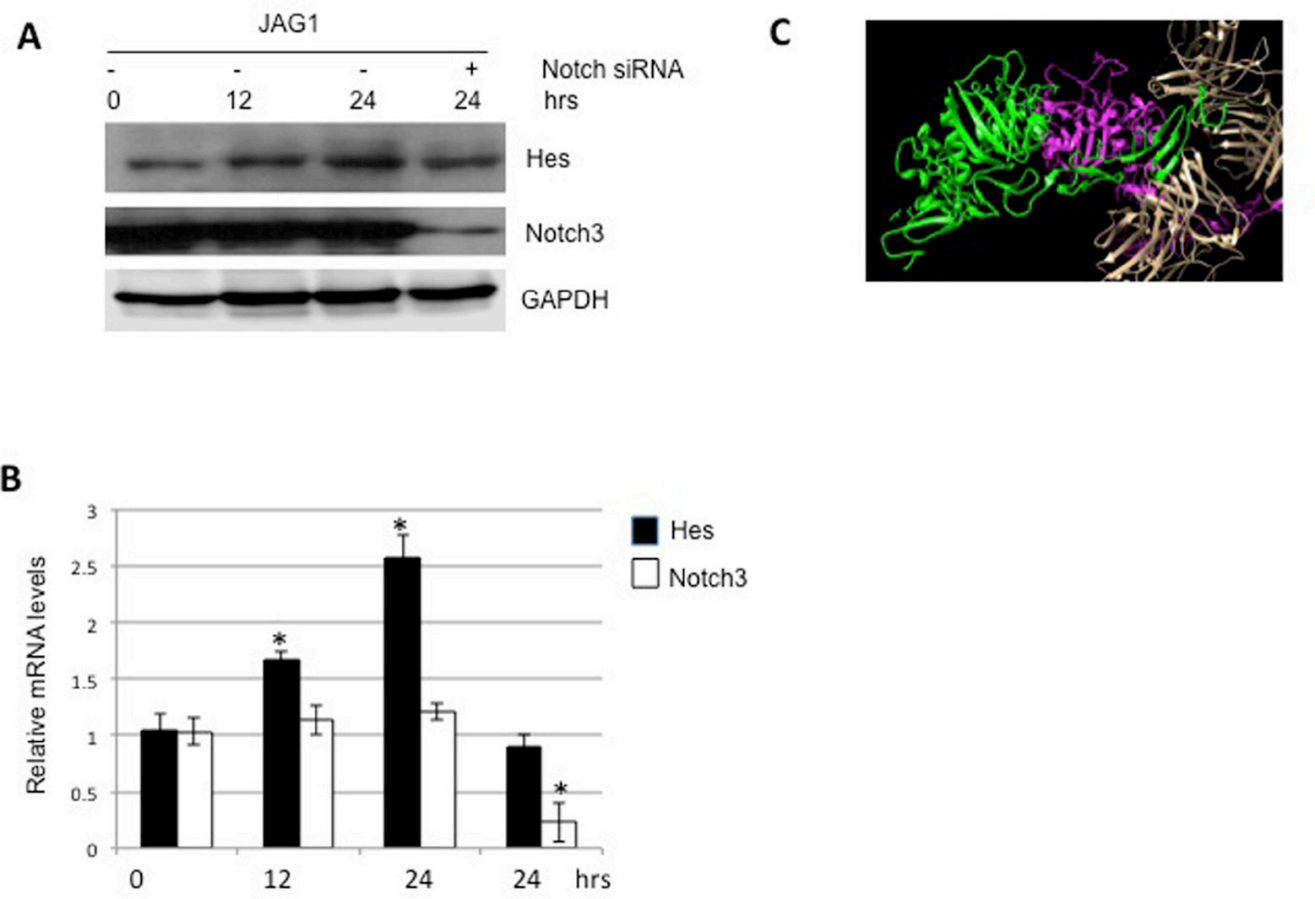
JAG1-Notch3 stimulates expression of Hes Frozen LS180 cells were defrosted and cultured in RMPI1640 and 10% FBS for 24 h. The media was changed to fresh one and the cells were , treated with JAG1 for the time periods as indicated. The whole lysates were subjected to Western blot analysis (**A**) or extracted for isolation of total RNA, followed by RT-qPCR analysis (**B**). (**C**) Interaction of JAG1 (magnate and green) and was Notch3 predicted with use of protein-protein docking webserver as described in Material and Methods. *N* = 3. ^*^ *P* < 0.01.

The canonical JAG/Notch signaling is activated to stimulate the transcription of Hes in a variety of cancers [8]. In order to confirm its role on colon carcinoma, we treated LS180 cells with JAG1. As shown in Figures 4A and 4B, JAG1 stimulated the production of Hes at both protein and mRNA levels. Moreover, the depletion of Notch3 abolished such stimulation. Structural interaction of APL13 and APJ was reported previously [21] (Figure 4C). These data suggest that JAG1/Notch3 signaling pathway is activated in colon carcinoma.

### Inhibition of APJ or Notch3 prevents APL13 or JAG1-mediated proliferation of colon carcinoma

**Figure 5:**
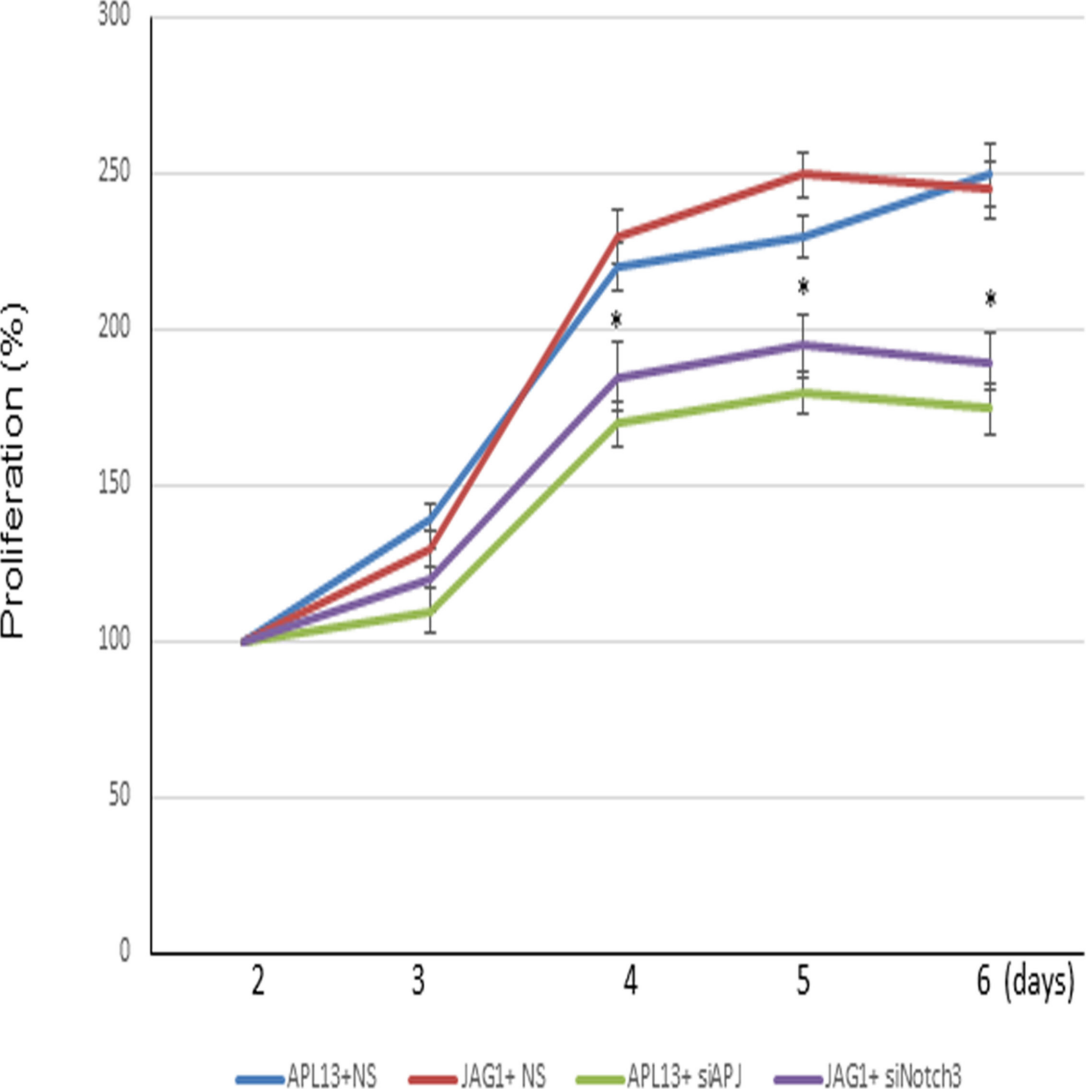
Inhibition of APJ or Notch3 reduces the LS180 cell proliferation Proliferation curves of cells treated with control siRNA (NS) or siRNA to APJ (siAPJ) or Notch3 (siNotch3). ^*^ *P* < 0.01.

We used siRNA-mediated gene silencing technique to assess these effects. LS180 cells were treated for 48 h with siRNA specifically targeting mRNA degradation of APJ or Notch3, and subsequently treated with APL13 or JAG1, respectively. As shown in Figure 5, depletion of APJ or Notch3 abolished APL13 or JAG1-associated proliferation of colon carcinoma. The results support a critical role of APL13/APJ-Notch3 signaling in proliferation of colon carcinoma.

## DISCUSSION

**Figure 6:**
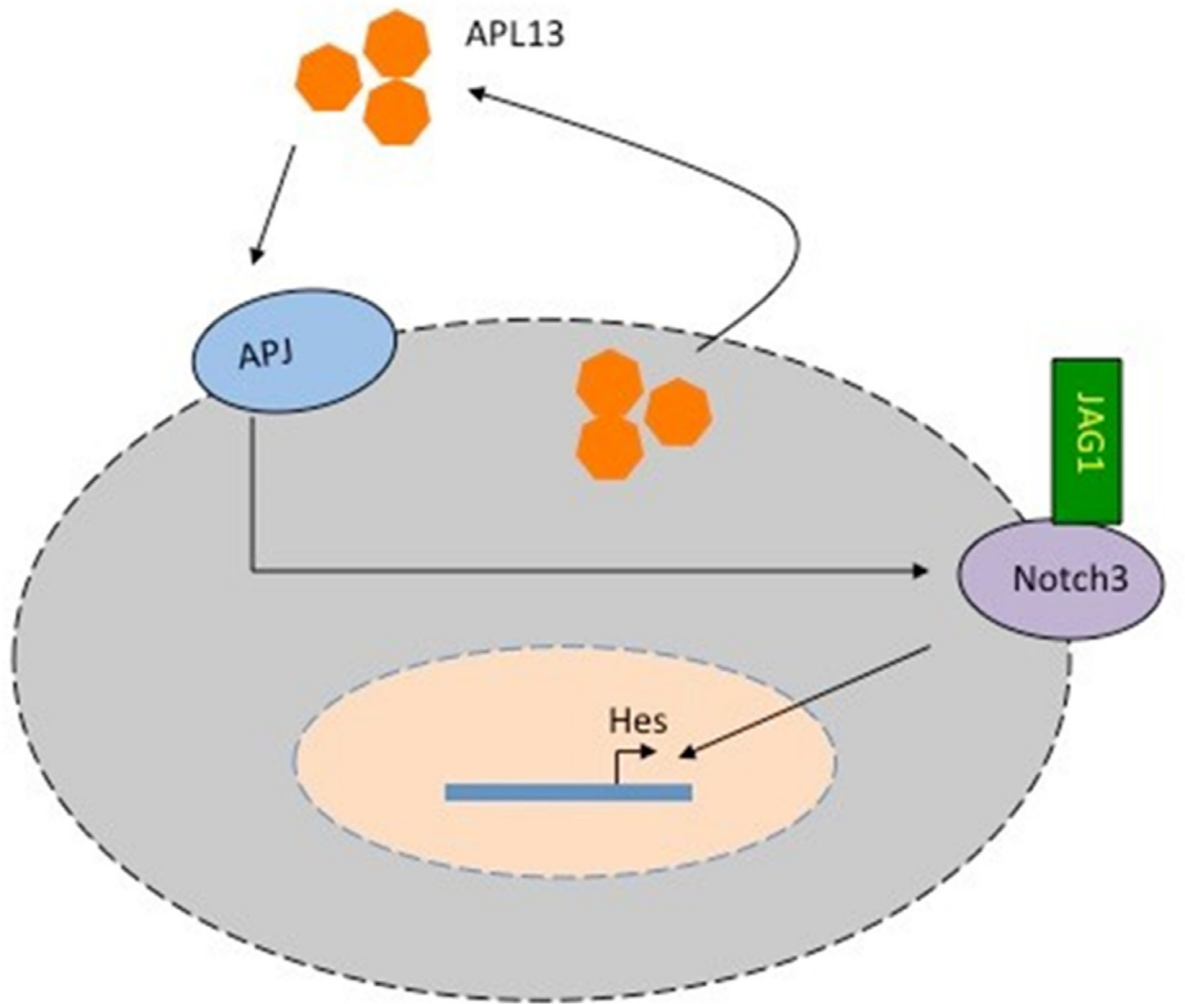
Model of APL13/APJ-Notch3 signaling in colon adenocarcinoma APL13 can be secreted from colon carcinoma cells and act on APJ in an autocrine manner. Along with exogenous APL13, APL13/APJ system upregulates Notch3, allowing exogenous JAG1 to bind to it. Activated JAG1-Notch3 signaling activates Hes and other genes promoting the proliferation of colon carcinoma cells.

APL13/APJ system has been found to be activated in a variety of cancer types and other pathological conditions like myocardial infarction [7, 20, 22, 23] and ischemic stroke because of its capabilities to induce angiogenesis. In addition, a large body of evidence supports the notion that dysregulation of the Notch pathway plays a significant role in the progression of several malignancies [9, 16–18, 24]. Moreover, increased expression levels of JAG1 are associated with malignant progression and metastatic potential, recurrence and poor overall survival in multiple types of solid cancer such as prostate cancer, breast cancer, and gastric cancer. Recently, it has been reported that JAG1 induces the transition from epithelial to mesenchymal phenotype as well as cell growth. It was also found that enriched expression of JAG1 is regulated by various pathways and is linked to poor prognosis of CRC [9]. To our knowledge, our present study is the first one linking the activation of APL13/APJ system to JAG1/Notch3 signaling in CRC (Figure 6). Our study shows that APL13 can activate APJ in an autocrine manner, and the activation APL13/APJ signaling up-regulates Notch3, thus allowing further binding of JAG1 to Notch3.

In contrast to previous reports [20, 25], in this study we were able to detect APL13, APJ, and Notch3 in normal epithelial tissues, albeit at significantly lower levels than in colon adenocarcinoma. Picault and colleagues have shown that APL13/APJ are expressed at low levels in colon adenoma and adenocarcinoma, and the concomitant stage-dependent expression is also associated with the similar tumor localization [20]. Further, the authors demonstrated that lower expression levels of proteins correlate with the low expression of receptor gene in the LoVo colorectal cancer cell line. Based on the findings presented in this study, we believe that there might be some carcinoma cells invading the adjacent epithelial cells that could be barely detected by the naked eye. However, the critical finding in this study is that there is a positive correlation between the expression levels of APL13, APJ and Notch3, and the pathogenesis and proliferation of CRC. Another interesting finding of this study is that the expression of APL13 by CRC cells *per se* supports the existence of autocrine loop, as reported by Picault et al [20]. These results could explain why CRC cells are capable of proliferating for extended periods of time even in the absence of serum.

Notch family receptors represent a large single-pass type I transmembrane proteins. In mammals, there are four Notch receptors: Notch 1 to 4. The extracellular domain of Notch family members is comprised of up to 36 tandemly repeated copies of an epidermal growth factor (EGF)-like motif. Notch receptors strictly interrelate with the single-pass type I transmembrane ligands including JAG1-2 and Delta-like (DLL1, DLL3 and DLL4), which are expressed on neighboring cells [8]. Such restrictions allow the Notch pathway to regulate short-range intercellular interactions. The most commonly induced Notch target genes include the basic helix-loop-helix (bHLH) transcriptional repressors of the Hes/Hey family proteins [26–28]. In this study, we demonstrate that Notch3, but not other Notch receptors, was preferably stimulated by APL13/APJ, and activated JAG1/Notch3 promoted the transcription of Hes and proliferation of CRC, consistent with the aforementioned studies. Apart from inducing the expression of Notch3 in CRC, APL13/APJ has been implicated in pathological angiogenesis in many malignances, based on the ability to modulate multiple molecular factors/pathways, such as AKT, CXCR4, VEGF, and ANG. As a result, these mechanisms could contribute to carcinogenesis, progression and metastasis of CRC in a coordinated way. Nonetheless, our findings would make APL13/APJ system a more convincing therapeutic target for the treatment of CRC.

## MATERIALS AND METHODS

### Cancer tissues and cell cultures

Colon cancer tissues were collected after patients signed the informed consent. The protocol was approved by the Ethics Committee of Second Hospital of Jilin University. Human colon carcinoma cell lines LS180 and HEK293 were purchased from ATCC (USA). Cells were maintained in RPMI-1640 medium supplemented with 10% FBS at 37°C and 5% CO_2_.

### Antibodies and reagents

Antibodies used in this study included anti-APL13 (Antibodies On-line, GA, USA), Anti-APJ (EMD Millipore, USA), anti-Notch3 (Abcam, MA, USA), Anti-Hes (Abcam, USA), and GAPDH (Cell Signaling, USA). APL13 and recombinant JAG1 were purchased from Santa Cruz Biotech (TX, USA) and Abcam (USA), respectively. FlexiTube siRNAs to APJ or Notch3 were purchased from QIAGEN (CA, USA). APL13 and Secretin ELISA kits were obtained from Antibodies On-line (GA, USA). The following primers were purchased from IDT DNA (IA, USA): APJ forward/reverse primers (5′-GTCCACCCCCTACTGGATTT-3′/5′- AGCAGGAACCCAGCTCAGTA-3′) NOTCH3 forward/reverse primers (5′- ATCTTGGGGGCCTAAAGAGA-3′/5′-GACTGAGAGGGTGGGTGGTA-3′) NOTCH1 forward/reverse primers (5′- GAAGATGCTCCAGC AACACA-3′/5′- TCTGCTCCTCCCAAACTAGG-3′) Hes forward/reverse primers (5′- CTCTCTTCCCTCC GGACTCT-3′/5′-AGGCGCAATCCAATATGAAC-3′) and GAPDH forward/reverse primers (5′-GTCAGTGGTG GACCTGACCT-3′/5′-TGCTGTAGCCAAATTCGTTG-3′).

### Co-Immunoprecipitation

For immunoprecipitation of APJ, we conjugated anti-APJ13 antibody to Protein G beads (Roche, USA), as specified by the manufacturer. We added these beads to reactions, incubated them for 1 hr at 4°C with mixing, washed the beads three times with IP buffer (25 mM Tris, pH7.4, 150 mM NaCl and 0.01% Triton X-100) and subjected the washed beads to western blot analysis.

### Western blot

Tissues or cells were lysed with RIPA buffer (Sigma, USA) on ice for 5 min and subsequently centrifuged at 14,000 × g for 15 min at 4°C, and the supernatants were collected. Samples were mixed with LDS Sample Buffer (Life Technologies, USA) and boiled for 5 min. The proteins were quantified using Nanodrop 2000 spectrophotometer (Thermo Scientific, USA),. Equal amounts of proteins were separated by electrophoresis on 10% SDS-PAGE and transferred to polyvinylidene difluoride (PVDF) membranes (BioRad, USA). Membranes were blocked in 5% non-fat milk power in 10 mM sodium phosphate buffer (pH 7.2), 150 mM NaCl, and 0.1% Tween 20 (PBST) for 1 h, washed twice with PBST, and incubated with antibodies as indicated in 1% nonfat milk powder-PBST at 4°C overnight. Blots were washed 3 times with PBST, incubated with the appropriate horseradish peroxidase-conjugated secondary antibodies at a 1:5000 dilution in 1% nonfat milk powder-PBST, and developed using Immuno-Star HRP substrate (Bio-Rad, USA).

### Immunofluorescent staining

As recently described [29], LS180 cells were first fixed with 4% paraformaldehyde in PBS and permeabilized for 10 min in 0.5% Triton X-100 in PBS at room temperature. Cells were then washed with PBS and incubated with appropriate antibodies as indicated at room temperature for 1 h. Cells were washed with PBS containing 0.1% BSA and incubated with fluorophore-conjugated secondary antibodies (Alexa Fluor 488 goat anti-rabbit IgG or 532 anti-mouse IgG) for 1 h at room temperature. The cells were then counterstained with DAPI (Invitrogen, USA) and visualized with fluorescent microscopy.

### ELISA

ELISA was performed using APL13 and Secretin ELISA kits according to manufacturer's protocols.

### Quantitative real-time RT-PCR (RT-qPCR)

Total RNA was extracted from colon cancer tissues or LS180 cells with the use of Trizol (Invitrogen, USA). Reverse transcription was carried out with 3 μg of total RNA using a SuperScript First-Strand Synthesis System for RT-PCR kit (Invitrogen, USA). The resulting cDNA was subjected to real-time PCR using SYBR green dye (Qiagen, USA) on ABI 7300 real-time PCR machine (Applied Biosystem, USA), as previously described [29]. The fold increase was determined by the method of cycle threshold (CT) using the formula 2^-ΔΔCT^.

### Gene silencing

Freshly prepared LS180 cells were plated in 6-well culture plates in 3 ml of RPMI-1640 containing 10% FBS. After being maintained in culture for 24 h, each well was transfected with the complex of FlexTube siRNA-Lipofectamine 2000 (3 μl; Invitrogen) prepared following the manufacturer's instructions with a final concentration of siRNA at 75 mM. A non-targeting siRNA or non-specific RNA was used as a negative control. At 48 h after exposure to the targeting or non-targeting siRNA, whole-cell lysates were prepared for Western blot analysis or RT-qPCR.

### Protein docking

The PDB files of JAG1 and Notch3 were obtained from PDB Databank. The docking was performed using the online server (http://vakser.compbio.ku.edu/resources/gramm/grammx) [30].

### Cell proliferation assay

MTT [3-(4,5-dimethylthiazol-2-yl)-2,5-diphenyltetrazoliumbromide] - based assay was performed to quantify the level of cell proliferation, as previously described [31]. Cells were seeded in 96-well plates (4,500 cells/well in 200 μL medium) and incubated for 24 h at 37°C, 5% CO_2_. LS180 cells were transfected using Lipofectamine 2000 Reagent (Life Technologies). Cells cultured with complete medium were used as blank control. At the end of culturing, 20 μL of 5 mg/ml MTT (Sigma, USA) solution were added to each well, and the cells were incubated for additional 4 h at 37°C. Supernatants were removed and formazan crystals were dissolved in 150 μL of DMSO (Sigma-Aldrich, USA). Finally, OD was measured at 490 nm using multi-microplate test system (Victor3, MA, USA). In each assay, five parallel wells were made, and the results were calculated as the mean of minimum three independent experiments.

### Statistical analysis

Each experiment was repeated three times. Student t test and one-way ANOVA were used to compare 2 and ≥ 3 groups, respectively. *P* < 0.05 was considered statistically significant.

## CONCLUSIONS

The present study demonstrates a novel connection between JAG1/Notch3 and APL13/APJ system, and that their inhibition can effectively prevent proliferation of CRC cells. Our results suggest that targeting APL13/APJ or JAG1/Notch3 may help to design new therapeutics for treatment of patients with CRC.

## Abbreviations

ANOVA: analysis of variance
APJ: apelin receptor
APL: apelin
bHLH: basic helix-loop-helix
BSA: bovine serum albumin
CRC: colorectal cancer
CT: cycle threshold
DAPI: 4’,6-diamidino-2-phenylindole
*DMSO*: dimethyl sulfoxide
EGF: Epidermal Growth Factor
FBS: fetal bovine serum
HRP: horseradish peroxidase
MTT: 3-(4,5-dimethylthiazol-2-yl)-2,5-diphenyltetrazoliumbromide
NICD: Notch intracellular domain
OD: optical density
PBST: phosphate buffered saline with Tween 20
PVDF: polyvinylidene difluoride
RT-qPCR: real-time quantitative PCR
siRNA: small interfering RNA
*VEGF*: Vascular Endothelial Growth Factor
